# Neuroendocrine Neoplasms of the Breast: Current Insights and Future Directions

**DOI:** 10.1002/cnr2.70059

**Published:** 2024-11-25

**Authors:** Lei Jiang, Xinyuan Pan, Zhiqiang Lang

**Affiliations:** ^1^ Department of Pathology Yantai Yuhuangding Hospital Yantai Shandong China; ^2^ School of Clinical Medicine Shandong Second Medical University Weifang Shandong China

**Keywords:** diagnosis, management, neuroendocrine neoplasms of the breast, review

## Abstract

**Background:**

The breast neuroendocrine neoplasms (NENs) represent a heterogeneous group of tumors and account for less than 1% of all NENs. The 5th edition of the WHO Classification of Breast Tumors in 2019 introduced a more stringent definition for breast NENs including neuroendocrine tumors (NETs) (G1, G2) and neuroendocrine carcinomas (NECs) (small cell carcinoma, large cell neuroendocrine carcinoma). While the diagnostic criteria and treatment of breast NENs still have some unsolved issues.

**Objective:**

We aim to discuss the diagnosis, treatment progress, existing problems, and future development direction of breast NENs.

**Methods:**

We provide a comprehensive review and evaluation of the diagnostic criteria, pathological features, utilization of immunohistochemical markers, molecular characteristics, pathogenesis, clinical significance, treatment options for breast NENs.

**Results:**

(1) Under the new definition, "pure" breast NENs are extremely rare. (2) Breast NETs are graded according to Nottingham grading System. Grading criteria utilized in other systems for NENs, such as mitotic count, ki67 proliferation index, and necrosis, have not been applied to breast NENs. (3) The WHO has not yet acknowledged the existence of NET G3 clearly. (4) The expression of NE markers may differ in breast NECs. (5) The treatment of breast NENs is still based on IBCs‐NST, and without any difference from invasive carcinoma with NE features. (6) The prognosis of breast NENs remains unclear, while the prognosis for NECs is significantly poorer than invasive breast carcinomas of no special type (IBCs‐NST) has been confirmed.

**Conclusion:**

Strict adherence to criteria is the key to correctly diagnose breast NENs with the exclusion of metastasis from other sites. Further exploration is required to determine the tissue origin of breast NENs and understand the pathogenesis. Efforts are still needed to establish unified diagnostic criteria and a unique diagnosis and treatment consensus for breast NENs.

## 1 Introduction

The neuroendocrine neoplasms (NENs) are uncommon tumors originating from the distributed neuroendocrine (NE) cells throughout the body, thereby potentially affecting multiple organs and systems, with a predilection for the lung, gastrointestinal tract, pancreas, and other anatomical sites. The incidence of NENs in the breast is less than 1% of all NENs [1], and the proportion of NENs in malignant breast tumors ranges from less than 0.1% to 20% [2]. The inaccurate detection rate of breast NENs can be attributed to several factors. First of all, the lack of routine immunohistochemical staining for NE markers in invasive breast cancers partly contributes to the discrepancy in detection rates [3]. Additionally, due to the constantly changing diagnostic criteria for breast NENs, the inclusion criteria of the cases in the study reports are not uniform. Currently, the research on NENs is insufficient, and there is an absence of consensus on many aspects [4, 5]. This paper will provide a comprehensive review of the diagnostic criteria, pathological features, utilization of immunohistochemical markers, molecular characteristics, pathogenesis, clinical significance, treatment options for breast NENs, and discuss the diagnosis, treatment progress, existing problems, and future development direction of breast NENs.

## 2 Nomenclature Evolution of Breast NENs

In 1963, Feyrter and Hartmann first proposed the differentiation of breast cancer into NE subtypes based on silver staining of two cases of mucinous breast cancer, which initiated the process of comprehending NENs. In 1977, eight cases of breast carcinoid tumors were reported for the first time, demonstrating abundant silver particles in the cytoplasm that morphologically resembled carcinoids found in other organs. In 1982, “Carcinoid” tumors were identified in the breast as argentophilic carcinoma based on the presence of neurosecretion particles observed through silver staining and electron microscopy. After that, the positive expression of synaptophysin (Syn) and chromogranin A (CgA) were found. In 2001, another study initially proposed the diagnostic criteria for primary NENs of the breast, stipulating that more than 50% of tumor cells should express neuroendocrine markers. Subsequently, in 2002, they assessed the diagnostic and clinical significance of detecting neuroendocrine cells in breast tissue and advocated for independent classification of tumors predominantly composed of neuroendocrine differentiated cells.

The definition of breast NENs has been revised by the WHO classification based on many studies conducted at different time periods. In 2003, the 3rd edition of the WHO Classification of Breast and Female Reproductive System Tumors introduced a novel categorization for breast NENs, following Sapino's criteria. This classification defines breast NENs as a tumor entity characterized by epithelial‐derived tumors exhibiting morphological similarities to gastrointestinal and pulmonary neuroendocrine tumors (NETs), with over 50% of tumor cells expressing neuroendocrine markers (CgA and Syn). According to the morphological characteristics, grade and degree of differentiation, the tumor was divided into three subtypes: solid neuroendocrine cancer (NEC), small cell/oat cell carcinoma, and large cell NEC. In the 4th edition of the WHO Classification of breast Tumors in 2012 [6], the concept of “invasive carcinoma with NE characteristics” was proposed, which was defined as tumors with similar morphological characteristics to gastrointestinal and lung NE tumors and expressing NE markers to varying degrees, as well as some distinct subtypes of breast cancer. The revised definition eliminates the requirement of a 50% threshold for expression of immunohistochemical markers and encompasses a wider spectrum of tumor types compared to the previous version, which include highly differentiated NETs, poorly differentiated/small cell carcinoma NECs, invasive cancers with neuroendocrine differentiation, solid papillary carcinomas, and cellular mucinous carcinomas. The 5th edition of the WHO Classification of Breast Tumors in 2019 introduced a more stringent definition for breast NENs [2], eliminated distinct types of breast cancer that can be clearly diagnosed, and emphasized that the diagnosis of breast NENs can only be established with (1) there are morphological characteristics resembling gastrointestinal and lung NE tumors and (2) tumor cells exhibit diffuse expression of NE markers. The NE tumor component should account for over 90% of the tumor. “Pure” breast NENs exclusively consist of (1) NETs (G1 and G2 grades), and (2) NECs (small cell carcinoma and large cell NEC). These modifications contribute to enhanced accuracy in understanding and improved diagnostic reproducibility. The WHO classification also provides criteria for diagnosing invasive breast carcinomas of no special type (IBCs‐NST), or other specific types of cancer, when tumor components with NE histological features or NE markers immunostaining are present in ≤ 90% of the tumor. When the NENs components are present between 10% and 90%, it can be diagnosed as invasive breast carcinomas of no special type (IBCs‐NST) or other specific types of cancer, along with NETs/NECs. If the proportion of NE pattern is < 10%, the diagnosis should indicate IBCs‐NST (or other specific types of cancer) with focal NE pattern and specify the percentage in the report.

In view of the evolution of WHO definition of breast NENs, the following points should be noted: (1) First, breast NENs are clearly a combination of malignant tumors originating from the epithelial cells. (2) Under a strict definition, “pure” breast NENs are extremely rare. (3) Grading criteria utilized in other systems for NENs, such as mitotic count, ki67 proliferation index, and necrosis, have not been applied to breast NENs. Breast NETs are graded according to the criteria for IBCs‐NST. (4) In daily practice, it is more commonly observed that IBCs‐NST lacks the morphological characteristics of NENs; however, some cells do express NE markers. These cases should be diagnosed as invasive carcinoma with neuroendocrine differentiation, otherwise NENs. (5) Although WHO requires diffuse expression of NE markers in tumor cells, the expression of these markers may differ in NEC, and therefore diagnosis should still be based on histological morphology and immunohistochemical expression. (6) The perspectives on NECs in different editions of WHO exhibit relative consistency, while controversies still exist regarding NETs. Some researchers even raise doubts about the existence of breast NET due to its lack of a clear definition, overlap with IBCs‐NST in terms of histological morphology, treatment and prognosis, and absence of similar molecular characteristics found in NETs occurring in other organs. It is deemed more appropriate to consider breast NETs as a subtype of IBCs‐NST [7]. Additionally, some researchers have also conducted classification studies on breast NETs based on G1, G2, and G3 (G3 means NEN with intermediate nuclear grade, high mitotic and no similar to pulmonary NEC morphologically) [8, 9]. However, the WHO has not yet acknowledged the existence of G3 clearly. One study even proposed an alternative classification method that differs from the 5th edition of WHO [4]. (7) It remains uncertain about the status of NEC between small cell carcinoma and large cell carcinoma (Table 1).

**TABLE 1:** Pathological diagnostic criteria of breast NENs.

		Histological structure	Cell morphology	Nottingham grading system			
				Structure level	Nuclear level	Number of nuclear divisions	Total points
NET	G1	Most of them are nidular and trabecular structures, but also papillary, island‐like, and acinar structures, and the interstitium is separated by thin fibrous vessels.	Fusiform, plasmacytoid, or polygonal cells with eosinophilic granular cytoplasm, or large clear cells.	3 or 2	1	1	≤ 5
	G2			3 or 2	2	1 or 2	6–7
	G3*			3	2	3	8
				Other histological features			
NEC	SMCC	Small cells of uniform size are densely distributed and grow infiltratively.	The cell boundary is unclear, the cytoplasm is sparse, the nucleus is deeply stained, the nucleolus is high, and the nucleolus is not obvious (the cell size of SMCC is less than three lymphocytes).	Nuclear division is common and focal necrosis is common. Carcinoma in situ with the same cell morphology was observed, and intralymphatic thrombus was common.			
	LCNEC	Tumors with NE morphology (organoid, nesting with palisading, trabeculae, rosettes).	The cell size was large, the cytoplasm was abundant, the nucleus was significantly polymorphic and the chromatin was rough.				

*Note:* NET G3*: Some researchers have defined NENs with medium nuclear grade and high mitotic count that are not similar to NEC as NET G3, but this subtype has not been clearly defined by WHO. Main diagnostic points of the LCNEC#: (a) Having the structural characteristics of NEC‐ at least in part. (b) Has the cytological characteristics of NENs. (c) The cell atypia exceeded NET G3. (d) It is very rare and must be diagnosed with sufficient evidence.

## 3 Clinicopathological Features of Breast NENs

### 3.1 Clinical Features

The clinical features of breast NENs were not significantly different from IBCs‐NST. This disease is more prevalent in postmenopausal or older women and can occasionally occur in men [10], without presenting clinical syndromes associated with ectopic hormone production, such as carcinoid syndrome. The age range at onset was 43–70 years, and the tumor size ranged from 1.3 to 5.0 cm (with an average size of 2.6 cm) [2]. Studies have indicated that small cell carcinoma tends to be diagnosed at a higher stage than other types of breast carcinoma, with approximately 40% showing regional lymph node metastasis [2].

The NENs of the mammary do not exhibit any distinctive imaging features, but typically manifest as solid masses with high density, irregular contours, and absence of calcification. It has also been reported that the characteristic malignant radiographic features, such as irregular shape, burr‐like margin, suspicious calcification, and acoustic shadows behind the mass, are less frequently observed in breast NECs and more likely to exhibit benign radiographic features compared with breast cancers without NE characteristics. CT examination helps to detect metastases and rule out the possibility of metastasis to the breast from other sites. Isotope assays facilitate the assessment of NENs by evaluating the localization of well‐differentiated NETs using somatostatin receptor (SSTR) scintigraphy or positron emission tomography‐computed tomography (PET‐CT) with gallium‐68‐labeled somatostatin analogues, while 18F‐FDG PET‐CT is employed for poorly differentiated or high‐grade cancers [1].

### 3.2 Histogenesis

There are two opinions about the histogenesis of breast NENs. One assumes that breast NENs originates from the neoplastic transformation of “normal” and/or “hyperplastic” NE cells within normal mammary glands. The other, more widely accepted perspective suggests that NE and epithelial cell differentiation take place during the multidirectional differentiation of tumor stem cells in the early stages of tumorigenesis. The evidence supporting the latter includes the lack of benign NET cells, the existence of clonal correlation between NE cells and malignant epithelial cells, as well as the clonal correlation between poorly NEC and intraductal components [11].

### 3.3 Pathological Characteristics

**FIGURE 1:**
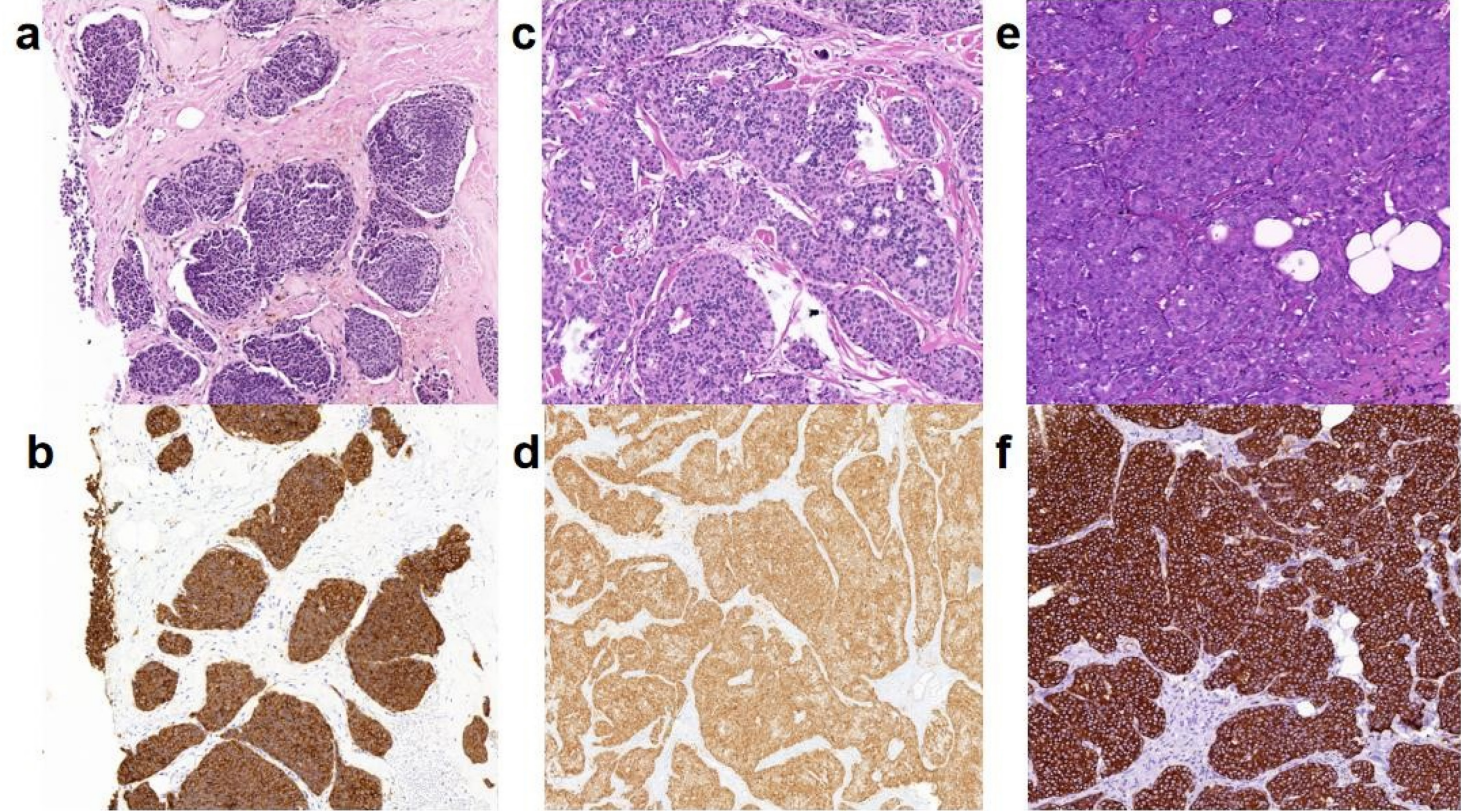
Well‐differentiated neuroendocrine tumor of the breast classed G1 (a), G2 (c), and G3 (e) with strong immunostaining of Syn (b, d, f).

The histological features of NETs and NECs in breast NENs are described in the 5th edition of the WHO Classification of Breast Tumors. NETs are aggressive neoplasm with NE differentiation, characterized by exhibiting dense cells arranged in nests and trabecular structures separated by thin fibrovascular stroma. These cells are fusiform or polygonal with eosinophilic granular cytoplasm, plasmtoid appearance, or large transparent morphology. Additionally, papillary, island‐like, and acinar structures may be observed; however, typical features of pulmonary carcinoids or gastrointestinal/ pancreatic NETs such as ribbon‐like cords and daisy clusters are not necessarily present [2]. The classification criteria for NETs in other systems are not applicable to the breast, but mitotic count remains the main parameter affecting grading. According to the Nottingham grading system, the majority of breast NETs should be classified as G1 or G2 (Figure 1). The diagnosis of breast NETs should primarily exclude metastatic NETs and moderately differentiated NECs. Distinguishing NETs from other breast tumors exhibiting neuroendocrine features is also necessary. Furthermore, breast NETs often represent mixed neuroendocrine and non‐NETs, which pose a significant challenge for the diagnosis.

**FIGURE 2:**
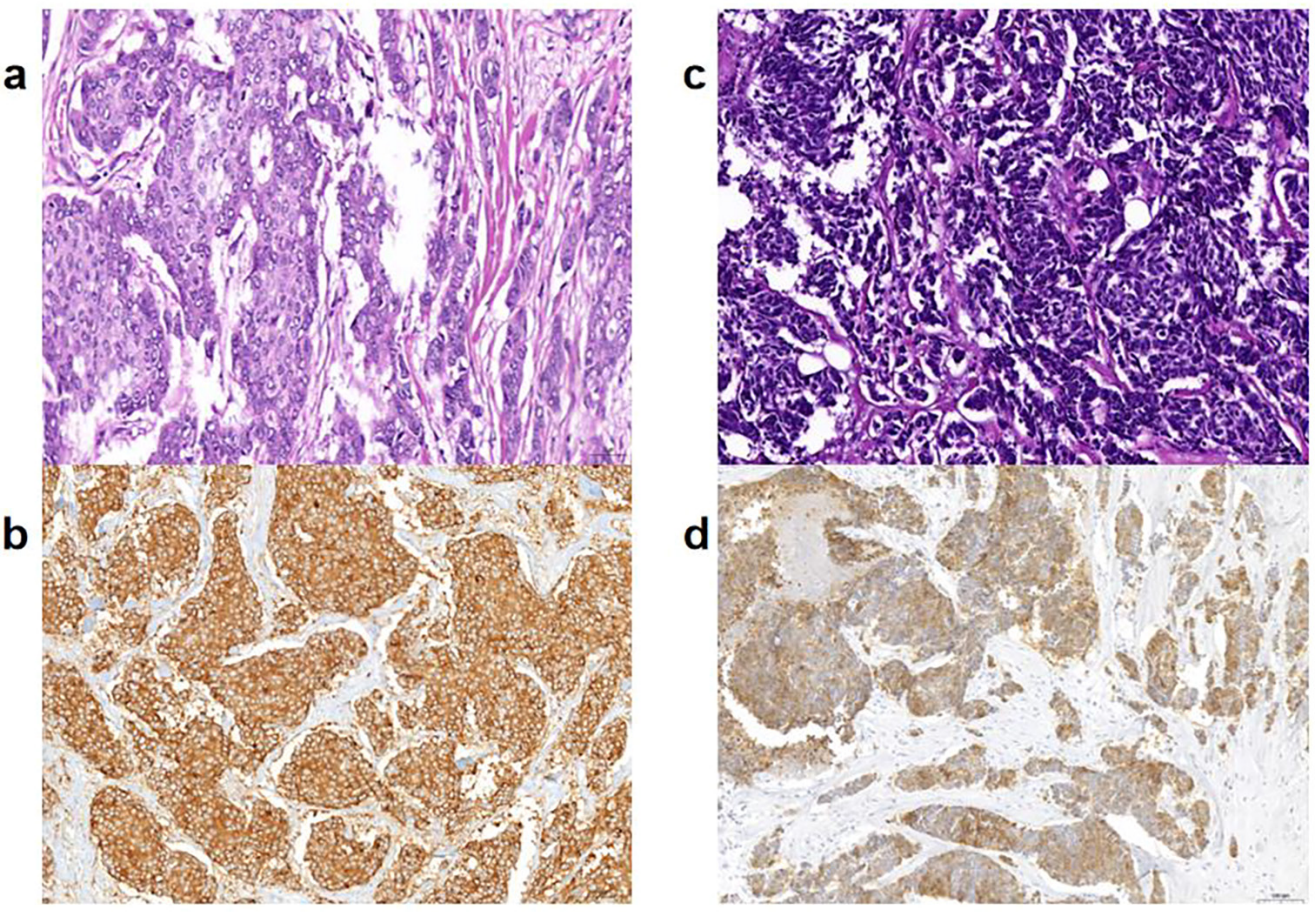
Large cell NEC (a) and small cell carcinoma of breast (c) with immunostaining of Syn (b, d).

NECs should exhibit histological characteristics consistent with small cell carcinoma or large cell NEC of the lung. Small cell carcinoma demonstrates invasive growth, characterized by a dense distribution of uniformly sized small cells, indistinct cellular boundaries, sparse cytoplasm, high nuclear/plasma ratio, and indistinct nucleoli. Large cell NEC cells are voluminous with abundant cytoplasm, polymorphic nuclei, and coarse chromatin. Both types commonly display evident mitoses and focal necrosis (Figure 2). Additionally, ductal carcinoma in situ (DCIS) with similar morphology could be seen, and lymphatic emboli could frequently be found. The differential diagnosis includes metastatic NECs, Merkel cell carcinoma, lymphoma, melanoma, solid adenoid cystic carcinoma, metaplastic carcinoma, basal‐like breast cancer, and so forth [2]. In rare cases, mammary NENs may exhibit signet ring cell‐like features [12] and micropapillary features [13].

### 3.4 Immunohistochemical Staining

The traditional markers for NENs include Syn, CgA, neuron‐specific enolase (NSE), and neural cell adhesion molecules (CD56). Among these, Syn and CgA are considered more reliable markers. Specifically, Syn demonstrates significantly higher sensitivity compared with CgA. The use of NSE and CD56 is limited to screening rather than for diagnosing breast NE differentiation. The Insulinoma‐associated protein 1 (INSM1), a novel neuroendocrine marker, has been validated in recent years to exhibit high sensitivity and specificity across multiple sites of NENs. Furthermore, its expression in certain tumors shows prognostic and therapeutic significance. INSM1, a 510‐amino acid protein, plays a crucial role in the regulatory transcription of NE differentiation and development, and it is the sole NE marker for diagnosis with nuclear staining. Multiple studies have demonstrated that INSM1 exhibits lower sensitivity compared with Syn but higher than CgA in breast NENs, while maintaining the same specificity as CgA and Syn [14]. It is worth noting that there are different interpretations for the expression of INSM1 in terms of intensity and percentage between researchers, further studies to establish standardized guidelines are needed. The SSTRs are G‐protein‐coupled receptors that could express in NETs of the lung, gastrointestinal tract, prostate, and other organs. Recent studies have demonstrated that, apart from SSTR1, breast NENs also exhibit positive expression of the remaining SSTR2A, SSTR2B, SSTR3, SSTR4, and SSTR5. Among these subtypes, positive staining of SSTR2A is predominantly found in breast cancer with a positive rate of 71% in NECs [15].

### 3.5 Metastasis

In addition to axillary lymph node metastasis, distant metastasis can occur in breast NECs, with the most common sites of bone, liver, lung, pancreas, soft tissue, pleura, and brain. A case study documented a 50‐year‐old patient with NECs who underwent left modified radical mastectomy, followed by whole breast radiotherapy and incomplete adjuvant chemotherapy until the development of leptomeningeal metastases. Subsequently, whole brain radiotherapy was administered along with a first‐line rescue regimen of etoposide and cisplatin. Unfortunately, 2 months later, the patient succumbed to leptomeningeal metastasis [16].

### 3.6 Differential Diagnosis

The diagnosis of primary NENs in the breast must exclude metastasis of NENs originating from other sites. Metastatic NENs account for only 1%–2% of metastatic tumors found in the breast. Primary and metastatic NENs may exhibit similar histological morphology. A retrospective study reported that up to 44% (8/18) of metastatic NENs cases were initially misdiagnosed as primary breast tumors [17]. The main distinctions between the two groups are as follows: (1) The presence of DCIS provides compelling evidence supporting primary NENs originating from the breast. (2) The existence of nuclear atypia or pleomorphism in well‐differentiated tumors substantiates the diagnosis of primary breast NENs. (3) The expression of immunohistochemical markers such as TRPS1, GATA3, and mammaglobin derived from the breast is helpful for the differential diagnosis [18]. While TRPS1 and GATA3 exhibit high positive rates in NETs, both demonstrate significantly higher negative rates in NECs. Consequently, distinguishing between NECs becomes more challenging. The digestive tract original marker CDX2 can be used to rule out the possibility of primary breast NENs according to its negative expression. However, the pulmonary marker TTF1 can also be positively expressed in poorly differentiated NENs of the breast, which has limited differential value. In view of the above limitations, a thorough check‐up is necessary.

### 3.7 Molecular Characteristics of Breast NENs

Due to the limited cases of “pure” breast NENs, there is a scarcity of relevant molecular research data. Targeted sequencing analysis has identified several genes, including FOXA1, TBX3, GATA3, and ARID1A, with a high frequency of mutations in breast NENs. Copy number alterations such as 8p11.23‐11.21, 8q24.12, and 8q24 are observed; however, the incidence of 1q gains and 16q losses is significantly less compared with ER‐positive IBCs‐NST [19].

At the transcriptional level, breast NENs are equivalent to the luminal subtype of breast cancer, with luminal B type being more prevalent, accounting for 71.26% [20], which exhibit a similar transcriptional composition to type B mucinous carcinoma and display significant upregulation of ESR1, FOXA1, and BCL2. Breast NENs also display higher mutation rates in chromatin remodeling genes ARID1A and ATRX compared with NENs found in other sites [21].

The genomic studies have revealed both similarities and distinctions in gene alterations between breast small cell carcinoma and lung small cell carcinoma. Both of them harbor TP53 mutations, and breast NEC exhibits the characteristics of both TP53 and RB1 alterations. Alterations in the PIK3CA/PTEN pathway and amplification of ZNF 703 are detected in 46% of breast small cell carcinomas [8]. Another next‐generation sequencing (NGS) study revealed a higher incidence of PIK3CA mutation in breast small cell carcinoma compared with lung small cell carcinoma, while no RB1 mutation was identified in the former [22]. The genetic disparities between these two diseases suggest distinct molecular mechanisms underlying each. Of the 11 recently reported TNBC with NE differentiation (TNBC‐NED), 7 (64%) showed p53 abnormal staining, 6 (55%) had Rb protein loss, while 6 (55%) had p53/Rb co‐abnormal staining/protein loss. These findings suggest a significant association between TNBC‐NED and Rb protein loss as well as p53/Rb co‐abnormality [23]. A study revealed that KMT2C (17.6%) was the most frequently mutated gene in breast NENs, as determined through whole exome sequencing (WES). The NENs exhibit copy number variations (CNVs) characterized by amplifications of 8q, 11q, and 17q and deletions of 11q and 17q. These CNVs involve loss of heterozygosity (LOH) suppressor genes like ACE, tumor driver genes including GATA3, and MAP3K4 and PDE4DIP genes associated with tumorigenesis. The frequency of oncogenic/probable oncogenic gene mutations in PI3K pathway genes and MAPK signaling pathway genes is higher in NETs compared with NECs (50%:18.2%) [24]. The latest studies utilizing NGS have revealed higher tumor mutation burden (TMB) and tumor neoantigen burden (TNB) in poorly differentiated NEC versus well‐differentiated NET, indicating distinct molecular characteristics of both. Notably, LOH at human leukocyte antigen (HLA) and loss of germline homogeneity (GDI) were prevalent in NENs, accounting 39% and 36%, respectively. The investigation highlighted that high TMB or gene mutations in TP53, KRAS, and HRAS are the most frequently observed therapeutic indicators [25]. Another paper also discovered a higher TP53 mutation rate and metastasis rate in NECs compared with IBCs‐NST and NETs, supporting their genetic and clinical distinction [26].

The above findings indicate that breast NENs represent a cohort of tumors with genetic heterogeneity.

## 4 Treatment of Breast NENs

Due to the scarcity of cases and limited availability of randomized experimental data, there is currently no specific treatment protocol for breast NENs. The treatment strategy of breast NENs needs reference to IBCs‐NST based on TNM stage, molecular typing, and other prognostic or predictive factors.

### 4.1 Surgery

Surgery remains the primary treatment modality for early‐stage breast NENs, with surgical approaches based on tumor location and stage. Cases involving lymph node metastasis are managed similarly to IBCs‐NST. Depending on the specific surgical approach employed, either sentinel lymph node biopsy or axillary dissection is performed.

### 4.2 Chemotherapy

The sensitivity to chemotherapy for NETs may be comparatively lower, whereas NEC can be effectively treated with chemotherapy. Due to without optimal solution, taxanes, anthracycline, or platinum is often used in daily work. Poorly differentiated or small cell NECs are often treated with platinum‐based or etoposide‐containing regimens, whereas anthracyclines and/or taxanes are often used for other types of NECs. The latest study conducted also demonstrated that patients who received/paclitaxel or paclitaxel alone exhibited improved overall survival (OS) and disease‐free (DFS) compared with those who did not receive these specific regimens (*p* < 0.05) [27]. Chikuie et al. [28] reported a case of a 76‐year‐old female with mediastinal lymph node recurrence of breast NEC on hemodialysis, who received chemotherapy with 5‐fluorouracil, epirubicin, cyclophosphamide (FEC 100), and docetaxel (DTX). The patient exhibited a complete response to the treatment, resulting in a significantly prolonged survival time. Anyway, the choice of treatment for breast NENs should be based on various influencing factors, including molecular typing, tumor size, nuclear grade, age, lymph node status, special tumor type, and so on.

The available evidence regarding the use of neoadjuvant chemotherapy for breast NENs is limited. A study administered four cycles of neoadjuvant chemotherapy using the TEC regimen, which includes polyene paclitaxel, epirubicin, and cyclophosphamide to a 43‐year‐old patient with breast NEC. The results demonstrated a significant treatment response as evidenced by a decreasing of the Ki67 proliferation index from 40% to 10%. Subsequently, the patient underwent modified radical mastectomy [29]. Although the guidelines do not provide a clear recommendation, some researchers suggest that neoadjuvant chemotherapy may be considered for patients with locally advanced or inoperable breast NECs who have a tumor size larger than 5 cm and express a strong desire for breast conservation. However, it is important to individualize the choice of neoadjuvant chemotherapy and take into account the comprehensive assessment of biological characteristics and various risk factors for recurrence [30].

### 4.3 Hormone Therapy

The majority of breast NENs belong to the luminal subtype, making endocrine therapy (ET) a highly valuable treatment option [1]. According to ASCO guidelines, ET is recommended for hormone receptor (HR)‐positive invasive breast cancer (NOS) patients for a duration of 5–10 years [31]. The use of TAM as a monotherapy is commonly employed for premenopausal patients. However, in cases of metastatic breast cancer, the combination therapy involving aromatase inhibitors or fulvestrant along with CDK4/6 inhibitors has demonstrated significant improvements in PFS and OS compared with ET alone [1, 32]. Another study [33] demonstrated that the combination of everolimus and aromatase inhibitor therapy significantly enhanced PFS in patients with HR‐positive advanced breast cancer who had previous treatment with nonsteroidal aromatase inhibitors. Consequently, the utilization of everolimus in conjunction with exemestane may be considered for metastatic breast NENs.

The findings indicate that PIK3CA mutations are present in 7%–33% of breast NENs and that PI3K pathway oncogenes are more prevalent in breast NET, suggesting that targeting the PI3K/AKT/mTOR pathway can serve as a reliable therapeutic approach for patients with (HR+)/human epidermal growth factor receptor 2‐negative (HER2−) breast NENs, particularly those with NETs [34, 35].

Recently, it has been reported by some researchers that combining abemaciclib with ET may also be considered for high‐risk HR+/HER2− breast NEN patients [36].

Endocrine therapy for currently available are aromatase inhibitors, selective estrogen receptor modulators, selective estrogen receptor dispensing, CDK4/6 inhibitors, mTOR inhibitors, and PI3K inhibitors [1].

### 4.4 Targeted Therapy

The therapeutic biomarkers in 20 cases of breast NECs were analyzed by Vranic et al., which identified the predictive expression of antibody‐drug conjugates (TROP‐2 and FOLR1) and histone deacetylase (H3K36Me3) inhibitors, providing valuable support for targeted therapy [37].

The utilization of anti‐HER2‐targeted therapy can significantly confer benefits to patients with rare HER2‐positive breast NENs. A patient diagnosed with HER2‐positive breast NEC exhibited no signs of disease progression for 9 years chemotherapy, radiation therapy, intravenous trastuzumab, and ET [38]. For patients with HER2‐positive breast cancer, commonly used targeted drugs include trastuzumab, pertuzumab, TDM‐1, ADC drugs, and so forth, tyrosine kinase inhibitors such as lapatinib, neratinib, and tucatinib have also demonstrated significant efficacy. Additionally, T‐Dxd, a novel ADC drug for HER2‐low breast cancer can be used for HER2‐positive patients [39].

Although there is limited research on the efficacy of these drugs in breast NENs, they can still be considered for HER2‐positive breast NENs.

A latest study in 2023 conducted an analysis of clinically actionable targets in NENs and revealed that the most frequently observed therapeutic indicators are high tumor mutational burden (TMB‐H) or gene mutations in TP53, KRAS, and HRAS. These findings confirm the eligibility to immune checkpoint blockade (ICB) and targeted therapy [26]. Additionally, there are other targeted treatment modalities available such as Peptide Receptor Radionuclide Therapy (PRRT), which utilizes radiolabeled somatostatin analogues to target NENs expressing SSTRs.

The exact efficacy of PRRT (90Y‐DOTATOC and 177Lu‐DOTATOC) on NETs has been reported in two cases, but further evaluation is required to ensure its safety. However, the use of somatostatin analogues alone in the treatment of NETs has not demonstrated clear clinical benefit [1].

### 4.5 Radiotherapy

There are currently no relevant clinical trials on postoperative radiotherapy for patients with breast NENs. Radiotherapy to the chest wall or regional lymph nodes is still performed following the treatment protocol for IBCs‐NST. A study suggested that patients with NECs who received radiotherapy appeared to have an extended survival time [40]. However, another study did not observe this trend in patients with small cell carcinoma [41].

### 4.6 Immunotherapy

Immune checkpoint inhibitors are the established standard of care for PD‐L1‐positive TNBC; however, there is a paucity of clinical data pertaining to breast NENs. Studies have demonstrated that the expression rate of PD‐L1 in NECs and NETs is 31.6% and 0%, respectively [42]. Considering the presence of microsatellite instability and high mutation burden observed in NEC, it may be plausible to explore the utilization of immune checkpoint inhibitors in the management of breast NECs [43].

### 4.7 Other Treatment Options

Bevacizumab, PARP inhibitors, and Sacituzumab govitecan can also be utilized for the treatment of breast NENs. Sacituzumab govitecan is an antibody conjugate that specifically targets Trop‐2 and can be employed in patients with locally advanced or metastatic urothelial carcinoma who have previously received platinum‐based chemotherapy and PD‐1/PD‐L1 inhibitors. Research on the application of this drug in triple‐negative NEC has also shown promising advancements [44].

## 5 Prognosis of Breast NENs

Due to the evolving definition of breast NENs by the WHO, there is inconsistency in the findings of prognosis studies conducted during different periods. Most studies prior to 2010 suggested that the prognosis of breast NENs was comparable or even superior to IBCs‐NST, primarily due to inclusion of favorable prognostic subtypes such as solid papillary carcinoma and cellular mucinous carcinoma in earlier investigations. However, recent studies have increasingly demonstrated a poorer prognosis for NENs. The 5‐year disease‐specific survival (DSS) and OS rates for small cell NEC were 50.5% and 32.2% respectively, which were significantly lower compared with those of other highly differentiated NENs (74.0% and 62.4%) [45].

The Surveillance, Epidemiology, and End Results (SEER) database released in a study to analyze the DSS and OS rates of 361 cases of breast NENs. The results revealed that the DSS rates for NETs, NECs, and IBC‐NST were 63.39%, 46.00%, and 89.17%, respectively, and OS rates were 55.66%, 38.87%, and 83.17%, respectively. These results support that NENs have a poorer prognosis compared with IBC‐NST. It is worth noting that the prognosis of breast NET, although generally considered favorable due to their slow‐growing nature in other organs, is still worse than that of IBCs‐NST [46]. Additionally, the study of Lai also supported that the IBCs‐NST with NE features have a poorer prognosis compared to invasive breast cancer without NE features [47]. Another study reported 6 cases of metastatic primary breast NEC with a median progression‐free survival (PFS) of only 5.8 months. All patients received various chemotherapy‐based treatments [48]. Some researchers demonstrated that patients with NECs have shorter disease‐free survival compared with IBCs‐NST patients with similar age, size, grade, and estrogen receptor status [34].

The prognosis of breast NENs remains unclear, with no consensus reached so far. However, the majority of studies support that the prognosis for NECs is significantly poorer than IBCs‐NST.

There are multiple prognostic factors associated with breast NENs, including tumor size, HR expression, ki67 expression, pathological type, stage, grade, and lymph node status [49]. Recent advancements in NGS [23] have identified a gene combination (MLH3, NACA, NOTCH1, NPAP1, RANBP17, TSC2, and ZFHX4) as a novel prognostic indicator for NENs. Patients harboring mutations in any of these seven genes exhibit significantly poorer survival outcomes. In addition, this study supports that HLA LOH is an important prognostic biomarker in a subgroup of NEN patients.

The choice of treatment regimen also significantly impacts the prognosis of patients with breast NENs. A comparative study demonstrated that patients who received chemotherapy regimens containing anthracycline/taxane or taxane alone exhibited improved OS and DFS compared with those who did not receive these regimens (*p* < 0.05). However, among the Stage I patients who underwent chemotherapy, there was no significant difference in OS or DFS compared with patients who did not receive chemotherapy [27]. Therefore, further exploration is required for optimal treatment plan selection and stratified management in order to get maximum therapeutic benefits.

## 6 Summary and Prospect

Breast NENs, a rare subgroup of breast tumors, after several version of the continuing evolution of the WHO classification, currently, have made significant advancements in defining, diagnosing, treating, and prognosis. The 5th edition of the WHO has introduced a relatively “pure” NETs (G1, G2) and NECs (small cell carcinoma, large cell carcinoma), thereby enhancing diagnostic standardization. However, in routine practice, the diagnostic consistency and repeatability of breast NENs need to be further improved [50]. The treatment of breast NENs is still based on IBCs‐NST, and without any difference from invasive carcinoma with NE features. Consequently, establishing unified diagnostic criteria and a unique consensus on the diagnosis and treatment of breast NENs serves as the foundation for optimizing clinical outcomes among patients. In future clinical practice and research, it is recommended to focus on the following aspects: (1) standardizing diagnostic criteria and terminology for breast NENs, particularly in clarifying the grading criteria of NETs (Does NET G3 exist? How to differentiate NET G3 with NECs?). (2) Conducting clinical randomized controlled trials and basic research according to uniform standards will facilitate the establishment of a reliable consensus on diagnosis and treatment. (3) Further exploration is required to determine the tissue origin of breast NENs and understand the pathogenesis.(4) Targeted therapy based on molecular features may offer more precise treatment options for breast NENs. (5) Identifying reliable methods to differentiate breast NENs from metastatic NENs, as well as distinguishing NET/NEC from other tumors with NE differentiation. (6) Given the limited number of cases involving breast NENs, strengthening multi‐center cooperation is essential for conducting more comprehensive research.

## Author Contributions

**Lei Jiang and Xinyuan Pan:** writing – original draft preparation and editing. **Zhiqiang Lang:** writing – review and supervision. All authors have read and agreed to the published version of the manuscript.

## Conflicts of Interest

The authors declare no conflicts of interest.

## Data Availability

Data openly available in a public repository that issues datasets with DOIs.
